# Diagnosis and Management of Skin Toxicities in Systemic Treatment of Genitourinary Cancers

**DOI:** 10.3390/cancers17020251

**Published:** 2025-01-14

**Authors:** Deepro Chowdhury, Laura Chin, Roupen Odabashian, Ali Fawaz, Christina Canil, Michael Ong, Mark G. Kirchhof, Martin. Neil Reaume, Ana-Alicia Beltran-Bless, Marie-France Savard, David J. Tsoulis, Dominick Bossé

**Affiliations:** 1 Division of Oncology, Department of Medicine, University of Ottawa, Ottawa, ON K1H 8L6, Canada; 2 Division of Medical Oncology, Princess Margaret Cancer Centre, Toronto, ON M5G 2C4, Canada; 3 Division of Dermatology, Department of Medicine, University of Ottawa, Ottawa, ON K1H 8L6, Canada; 4 Department of Oncology, Wayne State University, Detroit, MI 48202, USA; 5 Cross Cancer Institute, University of Alberta, Edmonton, AB T6G 1Z2, Canada

**Keywords:** rash, renal cell carcinoma, urothelial carcinoma, prostate cancer, skin disorders, Stevens–Johnson syndrome, immunotherapy, genitourinary cancers

## Abstract

The landscape of available therapeutic options for treatment of genitourinary (GU) cancers is expanding dramatically. Many of these treatments have distinct, sometimes severe, skin toxicities including morbilliform, bullous, pustular, lichenoid, eczematous, psoriasiform, and palmoplantar eruptions. Pruritus and skin pigmentation changes have also been noted. This review aims to synthesize dermatologic events observed with antibody drug conjugates, poly (ADP-ribose) polymerase (PARP) inhibitors, androgen receptor pathway inhibitors, tyrosine kinase inhibitors, immune checkpoint inhibitors, and the combination of these agents used for the treatment of GU cancers. It provides a guide on diagnosis and initial management of these rashes for medical oncologists.

## Simple Summary

Recent advancements in systemic therapies for genitourinary cancers, including immunotherapies, targeted therapies, and antibody-drug conjugates, have improved patient outcomes but have also introduced new skin-related side effects. These skin toxicities vary widely in presentation, severity, and onset, ranging from mild rashes to severe conditions like Stevens–Johnson syndrome. Prompt recognition and management of these skin reactions are crucial for patient safety, tolerability, and efficacy of cancer treatment. This review highlights the most common skin reactions associated with these novel therapies and offers practical strategies for their diagnosis and management. Improved collaboration between oncologists and dermatologists is essential to enhance patient care and address the challenges in managing cancer treatment-related skin toxicities.

## 1. Introduction

Systemic therapy treatment options available to genitourinary (GU) oncologists have dramatically increased over the past 10–15 years. Antibody-drug conjugates (ADCs), targeted therapies, immunotherapies, androgen receptor pathway inhibitors (ARPIs), poly(ADP-ribose) polymerase (PARP) inhibitors, and combination therapies have transformed the treatment landscape of GU malignancies beyond chemotherapy. Several of these new treatments are associated with skin toxicities, which are often challenging to manage in an oncology clinic. These skin toxicities are notable for their unique presentations and variable frequency, severity, and onset. The complexity is further heightened when novel agents are used in combination.

Accurate diagnosis and prompt management of these skin toxicities are essential to ensure patient safety, tolerance, and to optimize efficacy of treatment whenever possible. Unfortunately, the reporting of dermatologic side effects in clinical trials often lacks both accuracy and precision. Skin toxicities are frequently grouped under vague categories such as “rash” or “cutaneous toxicity”, without providing specific diagnosis of the eruption. This lack of specificity makes it challenging to develop a pragmatic clinical approach to improve the management of these adverse events. As a result, skin toxicity has become a common issue in GU oncology clinics, where access to dermatology expertise can be limited, and information on how to identify specific reactions and manage them effectively is often limited.

This review is targeted towards medical oncologists and summarizes the diagnosis and management of skin toxicities associated with systemic therapies used for GU malignancies. We review both commonly encountered eruptions as well as rarer but potentially life-threatening ones. This review was limited to skin toxicities that were prominent in clinical trials, associated with significant case reports in the literature, or associated with mortality (i.e., severe cutaneous adverse reactions, SCARs) and excludes nail, hair, or purely mucosal conditions. We also included references to direct the reader to more in-depth reviews of different skin reactions.

## 2. Materials and Methods

The authors summarized the available information in both the manuscript and Supplementary Materials on skin toxicities associated with common and emerging therapeutic classes of medications used in the treatment of GU malignancies. Product monographs, PubMed, as well as materials published at ASCO and ESMO conferences were searched. When multiple trials for a given agent were available, dermatologic toxicities were presented as ranges based on studies in which they could be clearly attributed to that agent. A breakdown of skin toxicity was obtained from the result tab of each trial on clinicaltrial.gov whenever available. All drugs were also researched for SCARs in the FDA Adverse Events Reporting System (specifically, “Steven-Johnson syndrome (SJS), Toxic Epidermal Necrolysis (TEN), SJS/TEN overlap”, “Drug Reaction with Eosinophilia and Systemic Symptoms (DRESS)”, and “Acute Generalized Exanthematous Pustulosis (AGEP)”) [1]. See Supplementary Materials for full FAERS query results.

For emerging drugs, manuscripts containing the highest quality information were prioritized, including phase I or II trial results when phase III trials were not available. Additionally, case reports and series were utilized to supplement information on rare but severe skin toxicities, such as SCARs. Cytotoxic chemotherapy agents, including platinum compounds and taxanes, were not included in this review. Instead, the manuscript focuses on immunotherapy, ADCs, fibroblast growth factor receptor (FGFR) and vascular endothelial growth factor (VEGF) tyrosine kinase inhibitors (TKIs), ARPIs, mammalian target of rapamycin (mTOR) inhibitors, and hypoxia inducible factor 1 subunit alpha (HIF-alpha) inhibitors. To maintain focus on clinically relevant and up-to-date data, we did not conduct a systematic review covering all molecules used in GU oncology. Instead, we concentrated on current, emerging, and promising agents. Cytotoxic chemotherapy was excluded to allow clearer analysis of newer agents without the confounding factor of chemotherapy-induced skin reactions. For example, we focused on darolutamide monotherapy rather than darolutamide combined with docetaxel, as a rash in patients receiving both agents could conceivably have arisen from either drug. All images demonstrating different types of eruptions were used with patients’ consent.

## 3. Cutaneous Drug Reactions

The reported skin adverse events associated with GU oncology drugs can be categorized into types of eruptions, along with pruritus and pigmentary changes. Each type of eruption has unique features and often requires different management. Below we have provided a description of the cardinal features to direct the clinician toward this diagnosis. A summary of the presentation, investigation, management, and prognosis for each reported reaction is provided in Box 1, Box 2, Box 3, Box 4, Box 5, Box 6, Box 7, Box 8 and Box 9**.** The boxes also contain key references that the reader can use for further information on the reaction, when to refer to dermatology and to biopsy. Notably, while many diagnoses can be made clinically, skin biopsies are useful in any case where the diagnosis cannot be made morphologically, if there are morphologic mimickers, or if the diagnosis needs to be confirmed because doing so would change systemic therapy management, or because the treatment needed for a suspected reaction has significant risks.

**Figure 1:**
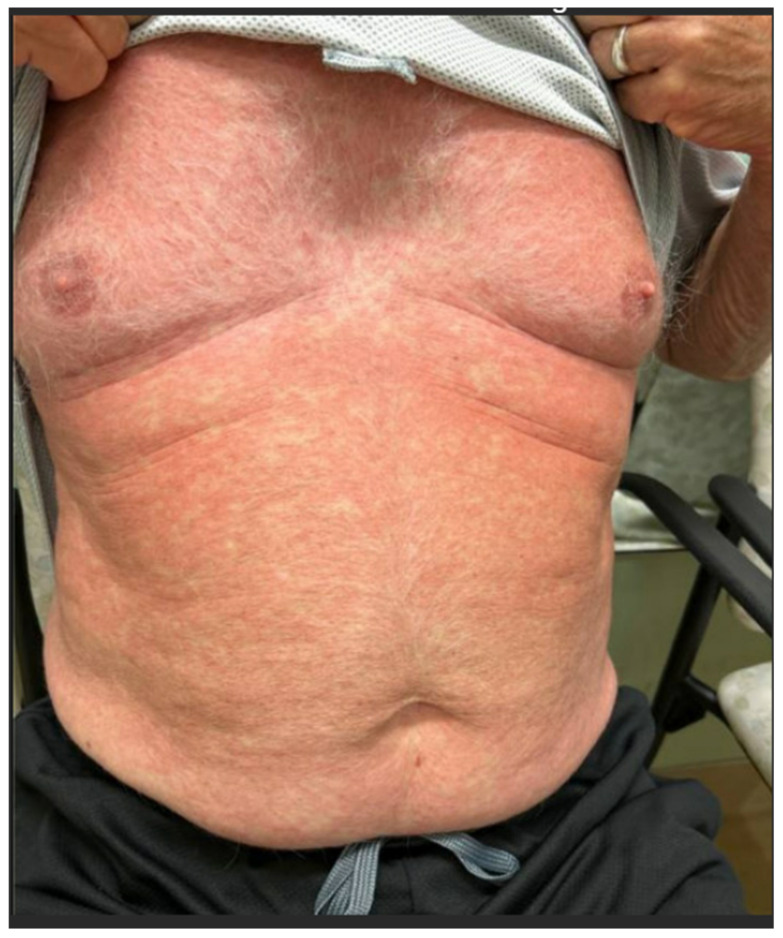
Morbiliform rash in a patient receiving apalutamide.

**Figure 2:**
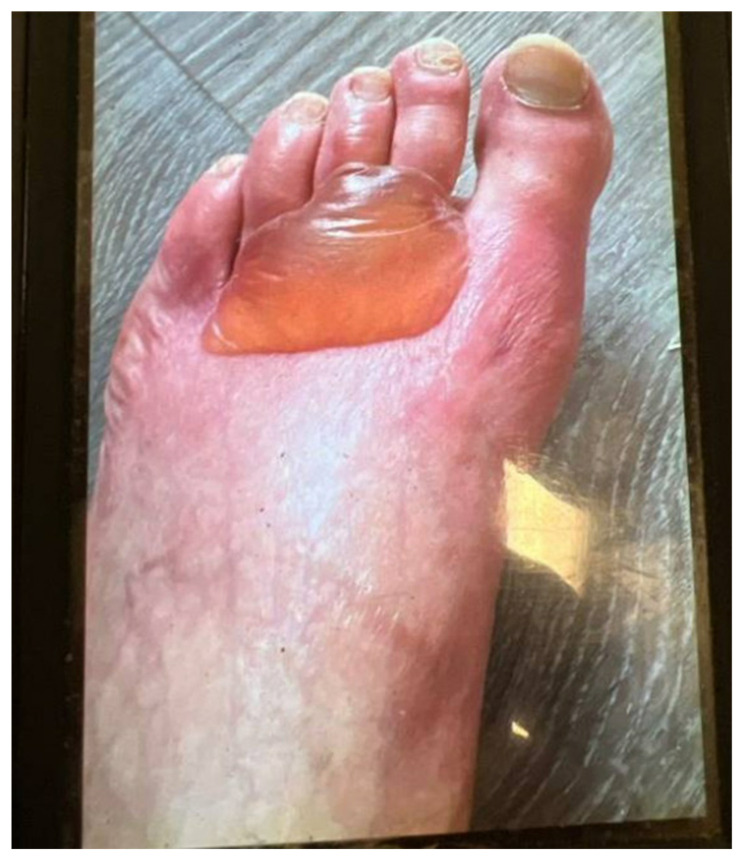
Bullous eruption in a patient receiving enfortumab vedotin.

**Figure 3:**
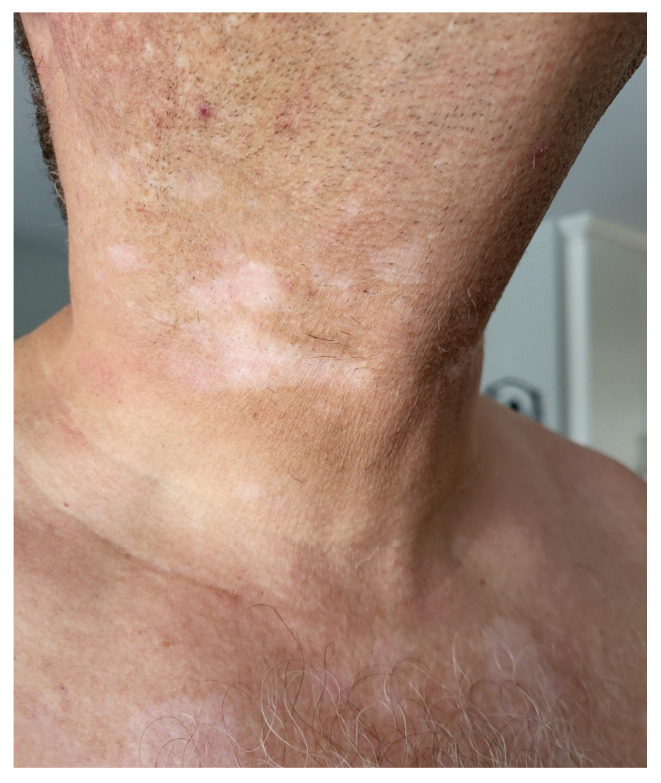
Vitiligo of the neck and chest in a patient receiving ipilimumab and nivolumab.

**Figure 4:**
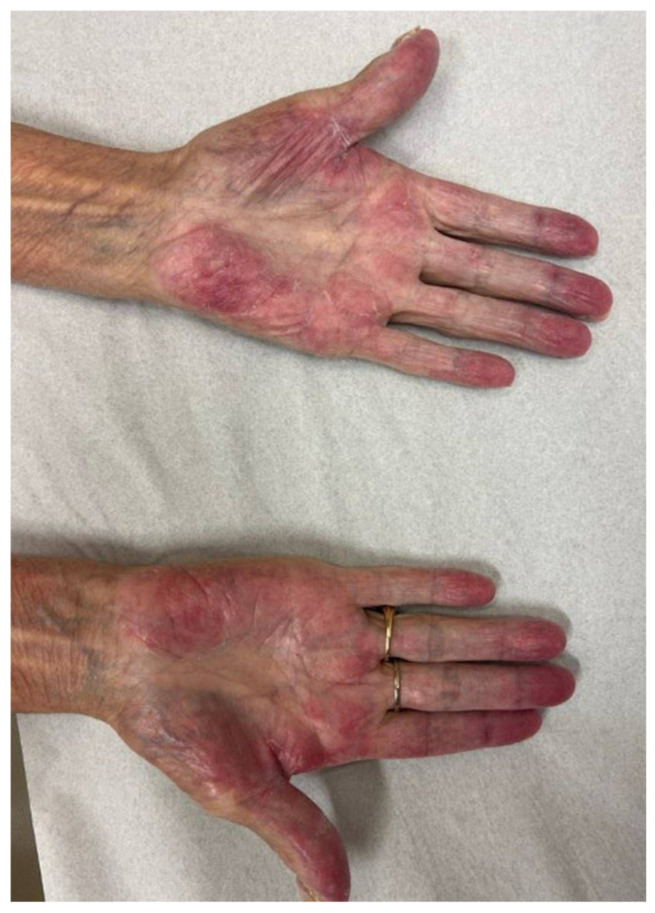
Palmar-plantar erythrodysesthesia in patient receiving erdafitinib.

- **Morbilliform (maculopapular) eruptions (Box 1):** Also referred to as “maculopapular”, morbilliform eruptions resemble measles, with widespread pink to red-brown macules and papules (see Figure 1). These lesions are usually blanchable but may become non-blanching in dependent areas (especially the legs but also in some cases the arms) [2]. Morbiliform eruptions may also be the initial presentation of a SCAR. Key warning signs include facial edema, peripheral eosinophilia, mucosal involvement, pustules, vesicles, blistering, exfoliation, painful or dusky lesions, erythroderma, or signs of systemic illness such as fever and lymphadenopathy [2].

- **Bullous (blistering) eruptions (Box 2):** Bullae are fluid-filled blisters larger than 1 cm in diameter. They can vary in appearance, being tense or flaccid, uni- or multiloculated, and surrounded by either normal or inflamed skin. Bullae may heal as erythematous, eroded patches, or plaques with collarettes of scale (see Figure 2) [2].

- **Pustular eruptions (Box 3):** Pustules are usually small (<1 cm), raised lesions filled with purulent fluid. These lesions can have either an inflamed, erythematous base, or a non-inflamed skin-colored base. They may coalesce into larger plaques of purulent collections and can be arranged in follicular or non-follicular patterns [2].
- **Lichenoid eruptions (Box 4):** Lichen planus is the prototypical clinically lichenoid eruption; however, many eruptions can share this clinical presentation. Classic lesions are discrete, deeply erythematous to violaceous, flat-topped papules and plaques, often with fine, lacy overlying scale known as Wickham striae. They can also occur on mucosal surfaces, appearing as macules, erosions, or ulcers [2].
- **Psoriasiform eruption (Box 5).** Psoriatic lesions are well-demarcated, erythematous papules and plaques with overlying silvery-white fine scale; psoriasis is the prototypical psoriasiform eruption [3,4,5].
- **Pruritus (Box 6):** Pruritus, or itching, can be associated with rashes or occur without visible skin changes [5,6]. If a rash is present, then treatment is usually targeted to that rash. It may be localized or generalized and can result from inflammatory conditions, organ dysfunction, nerve damage, psychogenic factors, or external irritants. Pruritus significantly affects quality of life and daily functioning [7,8].
- **Pigmentary Changes (Box 7).** Skin pigment changes can arise in association with various dermatoses, or as reactive changes following periods of skin inflammation, such as post-inflammatory hyper- and hypopigmentation [2]. The prototypical disorder of pigmentation is vitiligo (see Figure 3), an acquired autoimmune skin condition characterized by depigmented macules and patches due to loss of melanocyte function in the epidermis [5,6,9,10]. Lesions typically have a well-demarcated border, do not have associated scale, may koebnerize (lesions arising secondary to trauma), and may be preceded by inflammation and erythema [6,9,11]. Lesions are typically asymptomatic though early, inflamed lesions may have associated pruritus [11].

- **Palmar-plantar eruptions (Box 8).** Palmar-plantar erythrodysesthesia (PPE) is also referred to as hand-foot syndrome (HFS) and acral erythema. PPE is a well-known adverse effect from numerous traditional and newer systemic therapies. However, targeted multikinase inhibitors have been reported to cause a similar but distinct eruption [12,13]. Given the prominence of these eruptions in the oncology literature, we have separated them (see Figure 4).

- **Eczema (Box 9).** Classic eczematous lesions present acutely as pruritic, erythematous, and edematous with vesiculation with serous discharge and crusting. Chronically, eczematous lesions appear as pruritic, lichenified, papules, and plaques. Generalized xerosis, prurigo nodules, and excoriation may be seen [3].

Box 1 **Morbilliform (maculopapular) eruptions.**

**Morbilliform drug eruption (MDE)**
**Culprits: ICI:** nivolumab, ipilimumab, pembrolizumab, avelumab, durvalumab; **TKI**: sunitinib, pazopanib, sorafenib; **ADC**: enfortumab vedotin; **ARPI**: apalutamide. **Features: Onset**: **~**7–14 days [14,15]. **Presentation**: Pink to red-brown macules and papules often starting in axilla and groin, spreading and coalescing symmetrically across body. Often spares face and mucous membranes. Can be pruritic [5,6,14,15]. **Severity**: dose-dependent, may worsen with repeat treatment [6,10]. **Initial workup**: CBC with differential, liver enzymes, renal function; consider skin biopsy to rule-out SCAR (e.g., evolving DRESS, SJS/TEN). **Dermatology referral: Mild to moderate**: as needed. **Severe**: yes. **Initial management: Mild to moderate**: identify culprit drug; may treat symptomatically through course if necessary topical corticosteroids, supportive care: emollients, anti-itch topicals (e.g., containing pramoxine, menthol, camphor), antihistamines (if concomitant urticaria). **Severe**: discontinue culprit drug, systemic corticosteroids (~1 mg/kg/day with gradual taper) [5,6,14]. **Prognosis: Resolution**: 7–14 days [15].
**Drug reaction with eosinophilia and systemic symptoms (DRESS)/drug hypersensitivity syndrome (DHS)**
**Culprits: ICI:** nivolumab, ipilimumab, pembrolizumab, avelumab, durvalumab. **TKI**: cabozantinib, pazopanib, tivozanib, sorafenib. **ARPI**: abiraterone acetate, apalutamide, enzalutamide; ADC: enfortumab vedotin. **mTOR-inhibitors**: everolimus. **Features: Onset**: **~**2–6 weeks [16]. **Presentation**: can appear similar to MDE. Differentiating features: facial edema, follicular accentuation (inflammation of the hair follicles), and systemic features (e.g., fever, lymphadenopathy, arthritis/arthralgias, multi-organ involvement, peripheral eosinophilia) [14,17]. **Severity: variable.** **Initial workup**: Directed by patient presentation and directed to assess for diagnosis and internal organ involvement; could include CBC with differential, liver enzymes, renal function, chest X-ray (pulmonary findings in up to 25% [18]), troponin; consider skin biopsy. **Scoring system**: RegiSCAR, J-SCAR **Dermatology referral:** Yes, regardless of severity. **Initial management: Mild to moderate:** identify and discontinue culprit drug, topical corticosteroids, supportive care, such as emollients, anti-itch topicals (e.g., pramoxine, menthol, camphor), antihistamines (if concomitant urticaria). **Severe:** systemic corticosteroids (~1 mg/kg/day) with prolonged tapering course over months, consider cyclosporine as alternative (~3–5 mg/kg/day divided twice daily) [5,14,17]. **Prognosis: Resolution**: >14 days. S**equelae:** organ failure, autoimmune thyroiditis/Grave’s syndrome, SIADH, diabetes. **Mortality**: ~10%, most commonly related to organ failure (liver most common in classic DRESS; myocarditis often seen in ICI-related DRESS) [14,17].**Stevens**–**Johnson syndrome/toxic epidermal necrolysis (SJS/TEN) (early phase)****Culprits: ICI:** nivolumab, pembrolizumab, avelumab, durvalumab. **TKI**: axitinib, sunitinib, pazopanib, cabozantinib, lenvatinib, sorafenib. **ADC**: enfortumab vedotin. **ARPI**: apalutamide, abiraterone acetate, enzalutamide; mTOR: temsirolimus, everolimus. **PARP-inhibitors**: niraparib; olaparib. **Features: Onset: ~**7–21 days. Can be delayed up to 20 weeks with ICIs (new nomenclature for this delayed reaction: PIRME) [5,10,14,18,19,20,21]. **Presentation:** initial lesions may appear morbilliform or urticoid (raised, pruritic plaques—“hives”) which rapidly progress to dusky, gray to red-violaceous atypical targetoid lesions, then flaccid blisters with full-thickness skin sloughing involving both skin and mucous membranes (ex. mouth, eyes, urogenital region). Lesions are painful, **Nikolsky sign** (extension of blister with tangential pressure) and **Asboe-Hansen sign** (extension of blister with vertical pressure). Systemic features can include fever, malaise, upper respiratory tract and gastrointestinal symptoms [5,14,22,23]. **Severity: variable, often severe.** **Initial workup**: Directed by patient presentation; CBC with differential, liver enzymes, renal function, BUN, serum bicarbonate, glucose, chest X-ray, consider skin biopsy. **Scoring system**: SCORTEN **Dermatology referral:** Yes, regardless of severity. Also involvement of ophthalmology, and consider gyne/urology, and otolaryngology **services.** **Initial management: Mild**: discontinue culprit drug, can consider using high-potency topical corticosteroids (e.g., betamethasone valerate, clobetasol propionate, etc.), but have very low threshold for systemic therapy); oral ulcer treatment (e.g., triamcinolone acetonide paste); supportive care, such as fluid/electrolyte management, emollients, antihistamines (if concomitant urticaria), wound care, dressings; infection management. **Moderate to severe**:—cyclosporine (~3–5 mg/kg/day divided bid), etanercept or other TNF-alpha inhibitors (e.g., infliximab), **s**ystemic corticosteroids (~1 mg/kg/day with gradual taper); plasmapheresis and IVIg (~1–2 g/kg single infusion) are reported in the literature but not regularly used at our institution; consult other specialties for guidance on organ-specific management. **Prognosis: Resolution**: ~3 weeks [19]. **Sequelae**: slow-healing and scarring of skin and mucosa with resultant issues (e.g., ocular scarring leading to blindness, GI/GU scarring leading to strictures) [22,23]; secondary infections. **Mortality**: SJS ~10%, SJS/TEN overlap ~30%, TEN ~50% [5,10,14,22]. Most common cause of mortality is secondary infection leading to sepsis and multiorgan failure [24].

Box 2 **Bullous (blistering) eruptions.**

**Drug-induced bullous pemphigoid (DIBP)**
**Culprits: ICI:** nivolumab, pembrolizumab, ipilimumab, avelumab, durvalumab. **ADC**: enfortumab vedotin. **TKI**: cabozantinib, sunitinib, erdafitinib. **mTOR**: everolimus; ARPI: apalutamide; PARP-inhibitors: olaparib. **Features: Onset**: variable, days to months [6,14,25]. **Presentation**: tense, uni-loculated bullae, often preceded by pruritus and urticoid or maculopapular lesions. Lesions have normal skin-coloured bases with higher likelihood of palmoplantar and mucosal involvement; can mimic erythema multiforme or pemphigus +/− Nikolsky sign [6,10,14,25,26]. **Severity: variable.** **Initial workup**: CBC with differential, pemphigus and pemphigoid antibodies (at some institutions these are called “anti-skin antibodies”), skin biopsy with direct immunofluorescence. **Dermatology referral: Mild to moderate**: as needed. **Severe**: yes. **Initial management: Mild to moderate**: identify culprit drug; may treat symptomatically through course if necessary; high potency topical corticosteroids (e.g., betamethasone valerate, clobetasol propionate, etc.). Oral ulcer treatment (e.g., triamcinolone acetonide paste), dapsone, consider niacinamide, oral corticosteroids, supportive care: antihistamines, wound care, dressing, infection management. **Severe**: discontinue culprit drug, systemic corticosteroids (~1 mg/kg/day with gradual taper), methotrexate (10–25 mg weekly with folic acid supplement), rituximab, IVIg (~1–2 g/kg single infusion; may be repeated every 4 weeks), omalizumab, dupilumab [5,6,10,14]. **Prognosis: Resolution**: variable: days to months [6,10,14]; better prognosis than idiopathic BP [6,26].
**Stevens**
**–Johnson syndrome/toxic epidermal necrolysis (SJS/TEN) late phase**

**Refer to Box 1**

Box 3 **Pustular eruptions.**

**Acute generalized exanthematous pustulosis (AGEP)**
**Culprits: ICI:** ipilimumab, nivolumab, pembrolizumab, avelumab. **ARPI**: apalutamide, enzalutamide. **TKI**: sunitinib, pazopanib. **ADC**: enfortumab vedotin. **mTOR-inhibitors**: everolimus. **Features: Onset**: **~**24–72 h [27]; may be delayed. **Presentation**: acute onset of fever and non-follicular pustules on an erythematous, edematous base, favors truncal and intertriginous areas before becoming generalized, can be pruritic [27]. **Initial workup**: CBC with differential, liver enzymes, renal function, consider skin biopsy (to aid in differentiation from acute pustular psoriasis). **Dermatology referral:** Yes, regardless of severity. **Initial management: Mild to moderate**: discontinue culprit drug, high potency topical corticosteroids (e.g., betamethasone valerate, clobetasol propionate, etc.). Consider systemic corticosteroids; supportive care: emollients, antihistamines. **Severe**: discontinue culprit drug, systemic corticosteroids (~1 mg/kg/day with gradual taper) [27,28]. **Prognosis: Resolution**: rapid and spontaneous after drug discontinuation, mortality <5% [27].

Box 4 **Lichenoid eruptions.**

**Lichenoid drug eruption (LDE)**
**Culprits: ICI:** ipilimumab, pembrolizumab; nivolumab. **Interleukine**: IL-2; **ARPI**: apalutamide, enzalutamide. **Features: Onset**: delayed, average 15.7 wks after drug initiation [29]. **Presentation**: classically, pruritic violaceus flat-topped papules but can appear hypertrophic, papulosquamous, eczematous, bullous, or pustular. Common sites: trunk, limbs, acral; rarer sites: palmoplantar, nail, inverse, face/scalp, sun-exposed sites; more generalized distribution, less mucosal involvement, and less Wickham striae than idiopathic lichen planus [5,6,29,30,31,32]. **Severity: variable.** **Initial workup**: Consider skin biopsy (to aid in diagnosis and differentiate from idiopathic lichen planus). **Dermatology referral: Mild to moderate**: as needed. **Severe**: yes. **Initial management: Mild to moderate**: identify culprit drug; may treat symptomatically through course if necessary; topical calcineurin inhibitors (e.g., tacrolimus, pimecrolimus); topical corticosteroids; oral ulcer treatment (e.g., triamcinolone acetonide paste); supportive care, such as emollients, wound care, dressings; other dermatology-directed treatments: intralesional corticosteroids (e.g., triamcinolone acetonide). **Severe**: identify and discontinue culprit drug; systemic corticosteroids (~1 mg/kg/day with gradual taper); oral retinoids (e.g., acitretin or alitretinoin, 30 mg daily); methotrexate (10–25 mg weekly with folic acid supplementation); cyclosporine (~3–5 mg/kg/day divided bid); apremilast (gradual up-titration followed by maintenance dosing 30 mg daily or bid); phototherapy (nb-UVB); infliximab; tocilizumab [10,14,29]. **Note**: oral or genital lichen planus should be managed more aggressively to reduce risk of scarring [14]. **Prognosis: Resolution**: slow resolution, approximately 14 weeks [29]. **Sequelae**: post-inflammatory hyperpigmentation, scarring (especially mucosal).

Box 5 **Psoriasiform eruptions.**

**Drug-induced psoriasis**
**Culprits: ICI**: ipilimumab, pembrolizumab, nivolumab, avelumab. **ARPI**: apalutamide. **TKI**: lenvatinib. **Features: Onset**: **~**2–12 wks, shorter if history of idiopathic psoriasis [10,14,33]. **Presentation**: classic plaque psoriasis with bright pink-red papule and plaques with fine, silvery-white scale is common; other reported drug-induced variants include: guttate, pustular, palmoplantar, nail, inverse, erythrodermic [6,14]. **Initial workup**: Consider skin biopsy (to aid in diagnosis and differentiate from idiopathic psoriasis). **Dermatology referral: Mild to moderate**: as needed. **Severe**: yes. **Initial management: Mild to moderate**: identify culprit drug; may treat symptomatically through course if necessary; topical corticosteroids; topical PDE4 inhibitors; topical calcineurin inhibitors; topical vitamin D3 analogs (e.g., calcipotriol); topical retinoids (e.g., tazarotene); other dermatology-directed treatments: topical coal tar, phototherapy (nb-UVB), apremilast. **Severe**: identify and discontinue culprit drug; oral retinoids (e.g., acitretin 30 mg daily); methotrexate (10–25 mg weekly with folic acid supplementation), apremilast (gradual up-titration followed by maintenance dosing 30 mg daily or bid); biologics (IL-17, IL-23 inhibitors) [10,14]. **Note**: systemic corticosteroids should be avoided due to risk of disease flaring upon steroid withdrawal [14]. **Prognosis: Resolution**: highly variable.

Box 6 **Pruritus.**

**Drug-induced pruritus**
**Culprits:** ADC: enfortumab vedotin. **ICI**: ipilimumab, pembrolizumab, nivolumab, avelumab. **mTOR**: everolimus, temsirolimus. **TKI**: axitinib, sorafenib, pazopanib. **Features: Onset**: variable. **Presentation:** intense itching; may be associated with drug-induced xerosis or cutaneous eruption, but may also present in isolation [10]; secondary features of pruritus: erosions, ulcerations, nodules, lichenification [34]. **Initial workup**: Directed by patient presentation; skin biopsy usually not necessary unless associated with rash. Dermatology referral: as needed. **Initial management: Mild to moderate**: identify culprit drug; may treat symptomatically through course if necessary; topical anesthetics (e.g., pramoxine, lidocaine); topical corticosteroids; oral antihistamines; gabapentinoids; supportive care, such as emollients including compounded emollients with capsaicin, anti-itch topicals (e.g., containing pramoxine, menthol, camphor), wound care, dressings. **Severe**: identify and discontinue culprit drug; systemic anti-itch agents including gabapentinoids, aprepitant, naloxone, anticonvulsants, antidepressants; systemic corticosteroids (~1 mg/kg/day with gradual taper); phototherapy (nb-UVB); omalizumab; dupilumab [7,8,10,14,34]. **Prognosis: Resolution**: highly variable; may evolve into chronic pruritus.

Box 7 **Pigmentary changes.**

**Drug-induced vitiligo**
**Culprits: ICI**: ipilimumab, pembrolizumab, nivolumab, avelumab. **TKI**: sunitinib, sorafenib, pazopanib. **Features: Onset**: variable, ICI: ~9 months [35]. **Presentation:** flecked, depigmented macules that coalesce into patches in a bilateral, symmetric distribution; may have depigmentation of overlying hair (also known as poliosis) [6,10,14,34]; compared to idiopathic vitiligo, drug-induced vitiligo tends to have a more localized or photo-exposed distribution with less koebnerization [5,6,36]; fluorescence with Wood’s lamp; may be a positive predictive factor for tumor response to therapy [5,10,14]. **Initial workup**: Consider skin biopsy (for diagnostic confirmation and to differentiate from idiopathic vitiligo). Dermatology referral: As needed. **Initial management: Mild to moderate**: identify culprit drug; may treat symptomatically through course if necessary; topical corticosteroids; topical calcineurin inhibitors (e.g., tacrolimus, pimecrolimus); topical vitamin D3 analog (e.g., calcipotriol); topical Jak-inhibitors (e.g., ruxolitinib); supportive care, such as camouflage therapy with medical-grade makeup, sun-protection; other dermatology-directed treatments: phototherapy (nb-UVB). **Severe**: identify and discontinue culprit drug; IVIg (~1–2 g/kg; excimer laser; surgical modalities [14,34]. **Prognosis: Resolution**: highly variable; often evolve into chronic course [6,14,35].

Box 8 **Palmoplantar eruptions.**

**Palmoplantar erythrodysesthesia (PPE)/hand-foot syndrome (HFS)/acral erythema**
**Culprits: TKI**: sunitinib, pazopanib, cabozantinib, lenvatinib, erdafitinib, tivozanib, axitinib. **mTOR-inhibitor**: everolimus. **Features: Onset**: days to weeks [12,37]. **Presentation:** symmetric, erythematous, edematous, and tender lesions that may progress to blistering with desquamation, erosion, and ulceration; affects areas of friction, pressure, and other mechanical stress; pressure points on palms and soles, interweb spaces, lateral feet, elbows and extensor surfaces, amputated limb stumps [12,13]. **Initial workup**: Directed by patient presentation. Dermatology referral: As needed. **Initial management: Mild to moderate**: identify culprit drug; may treat symptomatically through course if necessary; topical keratolytics (e.g., containing salicylic acid, urea, etc.; high-potency topical corticosteroids (e.g., betamethasone valerate, clobetasol dipropionate, etc.); consider: oral vitamin E, vitamin B6; supportive care, such as emollients, pain management, wound care, dressings; preventative measures, such as patient information, avoidance of mechanical stress/friction/pressure/heat, regular emollient use, reducing sweat. **Severe**: identify and discontinue culprit drug; systemic corticosteroids (~1 mg/kg/day with gradual taper) [12,13,37]. **Prognosis: Resolution**: ~1–2 weeks following drug discontinuation [12,13].

Box 9 **Eczema.**

**Culprits: ADC + ICI**: enfortumab vedotin + pembrolizumab. **ICI**: pembrolizumab, nivolumab. **ARPI**: apalutamide. **TKI**: cabozantinib. **Features: Onset**: variable, weeks to months [37]. **Presentation:** also commonly referred to as “dermatitis” (skin inflammation), classic eczematous lesions may present acutely with pruritus, erythema, edema, and vesiculation with serous discharge and crusting and progress to generalized xerosis with pruritic, lichenified papules and plaques. Prurigo nodules and excoriations may be present [3]. **Initial workup**: Directed by patient presentation; consider skin biopsy or patch testing (to differentiate from idiopathic eczema or to identify contact dermatitis) [38]. **Dermatology referral**: As needed. **Initial management: Mild to moderate**: identify culprit drug; may treat symptomatically through course if necessary; emollients; topical corticosteroids; topical calcineurin inhibitors (e.g., tacrolimus, pimecrolimus); topical PDE-4 inhibitors (crisaborole); topical JAK-inhibitors (ruxolitinib); other dermatology-directed treatments: phototherapy (nb-UVB). **Severe**: systemic corticosteroids (~1 mg/kg/day with gradual taper); methotrexate (10–25 mg weekly with folic acid supplementation); biologics such as dupilumab (IL-4/IL-13 inhibitor), tralokinumab (IL-13 inhibitor); JAK inhibitors (JAK1: upadacitinib and abrocitinib; JAK 1/2: baricitinib (off-label)) as well as other systemic agents (cyclosporine, azathioprine, mycophenolate mofetil) are used in treatment of idiopathic eczema but may be contraindicated due to concurrent malignancy [38,39,40]. **Prognosis:** Variable.

Table 1 summarizes the incidence of “rash”, PPE, and pruritus reported in clinical trials for each anticancer agent used in GU oncology, along with their respective grades based on the Common Terminology Criteria for Adverse Events (CTCAE) [14]. The CTCAE grading system categorizes adverse events into five levels of severity:

**Grade 1**: Mild; asymptomatic or mild symptoms; clinical or diagnostic observations only; no intervention required.

**Grade 2**: Moderate; minimal, local, or noninvasive intervention indicated; limits age-appropriate instrumental activities of daily living (ADLs).

**Grade 3**: Severe or medically significant but not immediately life-threatening; requires hospitalization or prolongation of hospitalization; disabling; limits self-care ADLs.

**Grade 4**: Life-threatening; urgent intervention required.

**Grade 5**: Death related to the adverse event.

Notably, while the above CTCAE grading definition is broadly applicable to all adverse events, individual toxicities also have specific criteria: such as skin eruptions (e.g., rash maculopapular, acneiform, bullous dermatitis, etc.), which factor in the percentage of affected body surface area [grade 1, <10%; grade 2, 10–30%, grade 3, >30%] and the presence/absence of associated symptoms such as pruritus or tenderness [41]. CTCAE grading for maculopapular rashes, eruptions, and pruritus are provided in Appendix A.

In addition to the table, the text below offers further context for each drug, classifying rashes into specific types of eruptions. For management strategies based on eruption type, refer to Box 1, Box 2, Box 3, Box 4, Box 5, Box 6, Box 7, Box 8 and Box 9.

**Table 1:** **GU systemic therapies and associated rates of dermatologic toxicities.**

Therapeutic Class	Mechanism of Action/Target	Drugs	Skin Reaction(s)	All Grade/Grade 3–5	PPE All Grade/Grade 3–5	Pruritus All Grade/Grade 3–5	References
Antibody drug conjugate (ADC)	**Target**: Nectin-4 **Payload**: MMAE	Enfortumab vedotin	Maculopapular Rash Xerosis Drug eruption Hyperpigmentation Blister Dermatitis bullous Vesicular Toxic Skin eruption Xerosis SJS/TEN AGEP DRESS	19.2%/1.2% 18.5%/1% 16.9%/NR 10.1%/0.7%% 6.4%/NR 0.3%/0.3% 0.3%/0.3% 0.3%/0.3% 0.3%/0.3% 16.9%/NR FAERS FAERS FAERS	NR	32.1%/1.4%	[42,43,44]
ADC + immune checkpoint inhibitor (ICI)	**Target**: Nectin-4 **Payload**: MMAE ICI: PD-1 inhibitor	Enfortumab vedotin + Pembrolizumab	Maculopapular Xerosis Macular Papular Dermatitis Hyperpigmentation SJS/TEN Morbiliform Erythematous DRESS	34%/1.6% 17.3%/NR 9.7%/0.5% 7.7%/NR 6.3%/0.5% 5.5%/NR 1%/1% 0.5%/0.5% 0.2%/0.4% FAERS	NR	39.8%/1.1%	[45,46]
ICI	PD-1 inhibitor	Pembrolizumab Nivolumab	Rash Maculopapular Acneiform Erythema multiform Xerosis Lichenification Xerosis SJS/TEN DRESS AGEP	20–21.5%/0–0.3% 5.7%/0.2–0.5% 3.4%/NR 0.3%/0.3% 0–0.53% 0–0.2%/0–0.2% 0–0.53%/NR 0–0.2%/0–0.2% FAERS FAERS	0–5.43%/NR	22.7%/NR	[46]
	PD-L1 inhibitor	Avelumab, durvalumab	Rash Xerosis SJS/TEN DRESS AGEP	12.5%/NR 6.7%/NR FAERS FAERS FAERS	NR	18.6%/NR	[47,48,49,50]
	PD-1 inhibitor + CTLA-4 inhibitor	Nivolumab + Ipilimumab	Rash Maculopapular Xerosis Skin discoloration Drug eruption SJS/TEN DRESS AGEP	25.8%/0.4% 10.9%/0.4% 10%/NR 0.6%/NR 0.2%/0.2% 0.2%/0.2% FAERS FAERS	1.6%/NR	33.1%/0.2%	[47,51,52]
Androgen Receptor pathway inhibitor (ARPI)	Androgen receptor antagonist	Apalutamide	Rash Maculopapular Eczema Drug eruption Erythema Multiforme Xerosis Erythema Granuloma annulare SJS/TEN AGEP DRESS	10.8–20.2%/0–0.2% 3.2–5.3%/NR 2.3–0.12%/0–0.12% 0–0.2%/0–0.2% 0–0.12%/0–0.12% 0–3.4%/NR 0–2.7%/NR 0–0.12%/0–0.12% FAERS FAERS FAERS	NR	6.2–11.1%/NR	[53,54,55,56]
		Enzalutamide	Rash Xerosis Toxic skin eruption SJS/TEN AGEP DRESS	0–6.2%/0–0.11%/NR 0–6.2%/NR 0–0.23%/0–0.23% FAERS FAERS FAERS	NR	NR	[57,58,59,60,61,62,63,64,65]
		Darolutamide	Rash	1.8–2.5%/NR	NR	1.8–2.3%/NR	[65,66]
	CYP-17 inhibitor	Abiraterone Acetate + prednisone	Maculopapular Rash Erythema Vasculitis SJS/TEN DRESS	0–14%/NR 0–9%/NR 0–0.1%/0–0.1% 0–0.1%/0–0.1% FAERS FAERS	NR	NR	[67,68,69,70,71,72,73]
PARP inhibitors	trapping PARP1 and PARP2	Olaparib	NR	NR	NR	NR	[74,75,76,77]
		Rucaparib	Rash Photosensitivity Xerosis	7–7.9%/NR 6.9–10%/NR 6.5–8.2%/NR	NR	0–7.4%	[78,79,80,81]
		Niraparib	NR SJS/TEN	NR FAERS	NR	NR	[82,83]
		Talazoparib + enzalutamide	Rash Xerosis Maculopapular	3.8%/NR 3.3%/NR 1%/NR	NR	1.8%	[84,85]
Tyrosine Kinase Inhibitor (TKI)	VEGF receptor targeted therapy	Sunitinib	Discolouration (yellowing) Rash Xerosis Hypopigmentation Rash maculopapular Dermatitis acneiform Skin ulcer Urticaria Erythema multiforme Drug eruption SJS/TEN	8.4–15.2%/NR 8.1–22.3%/NR 4.7–9.9%/NR 1.3%/NR 0–5.23%/NR 0–0.9%/NR 0–0.3%/0–0.3% 0–0.3%/0–0.3% 0–0.2%/0–0.2% 0–0.2/0–0.2% FAERS	37.5–50.2%/0–0.2%	4.7–10.8%/NR	[46,86,87,88,89,90,91,92]
		Pazopanib	Rash Xerosis Hypopigmentation Discoloration SJS/TEN	17.3%/0.2% 7.8%/NR 4.9%/NR 0.7/NR FAERS	29.4%/0.4%	4%/NR	[88,89]
		Cabozantinib	Rash Xerosis Eczema Dermatitis bullous SJS/TEN DRESS	0–10.6%/NR 0–8.2%/NR 0–0.4%/0–0.4% 0–0.3%/0–0.3% FAERS FAERS	41–41.2%/NR	0–7.8%/NR	[93,94,95,96]
		Tivozanib	Rash Xerosis Erythema Maculopapular DRESS	9.9%/NR 6.4%/NR 1.7%/NR 0.6%/NR FAERS	16.2%/NR	2.3%/NR	[97,98]
	FGFR1–4 targeted therapy	Erdafitinib	Skin disorder ‡ Xerosis	54.8%/11.9% 23%/1.5%	30.4%/9.6%	NR	[99,100]
TKI + ICI	VEGFR targeted therapy + PD-1 inhibitor	Axitinib + Pembrolizumab	Rash Xerosis Maculopapular Drug eruption Skin ulcer SJS/TEN	14.2%/NR 6.8%/NR 0.2%/0.2% 0.2%/0.2% 0.2%/0.2% FAERS	28%/0.5%	15.2%/NR	[46,91]
		Lenvatinib + Pembrolizumab	Rash Maculopapular Xerosis Dermatitis acneiform SJS/TEN	27%/NR 8%/NR 6.3%/NR 1.4%/NR FAERS	28.7%/NR	14.5%/NR	[87,90]
		Cabozantinib + Nivolumab	Rash Maculopapular Xerosis SJS/TEN DRESS	21.6%/NR 8.4%/NR 6.6%/NR FAERS FAERS	40%/NR	18.8%/NR	[86,101]
hypoxia-inducible factor 2α inhibitor	hypoxia-inducible factor 2α inhibitor	Belzutifan	Xerosis Rash 4.6% Maculopapular	0–6.6%/NR 0–4.6%/NR 0–4.9%/NR	NR	4.9–7.8%/NR	[102,103,104]
Serine/threonine kinase inhibitor	mTOR inhibitor	Everolimus	Rash Xerosis Dermatitis acneiform Maculopapular Erythema Neutrophilic dermatosis Erythema multiforme SJS/TEN AGEP DRESS	0–30.3%/0–0.3% 0–15.7%/NR 0–12.6%/NR 0–6.1%/0–0.3% 0–5.5%/NR 0–0.3%/0–0.3% 0–0.3%/0–0.3% FAERS FAERS FAERS	5.9–9.57%/NR	0–16.1%/0–0.3%	[96,105,106,107,108,109,110]

Abbreviations: ADC, Antibody Drug Conjugate; AGEP, Acute Generalized Exanthematous Pustulosis; ARPI, Androgen Receptor Pathway Inhibitor; CYP-17, Cytochrome P450 17A1; DRESS, Drug Reaction With Eosinophilia And Systemic Symptoms; FAERS, FDA Events Reporting System; FGFR, Fibroblast Growth Factor Receptor; ICI, Immune Checkpoint Inhibitor; MMAE, Monomethyl Auristatin E; mTOR, Mechanistic Target Of Rapamycin; NR, Not Reported; PARP, Poly (ADP-Ribose) Polymerase; PPE, Palmar-Plantar Erythrodysesthesia; SCAR, Severe Cutaneous Adverse Reaction; SJS, Stevens–Johnson Syndrome; TEN, Toxic Epidermal Necrolysis; VEGFR, Vascular Endothelial Growth Factor Receptor. Skin reaction breakdown as posted on “Tab result” of clinicaltrial.gov for each respective trial referenced unless “case reports” specified. ‡ Skin disorders: blister, dry skin, erythema, hyperkeratosis, palmar erythema, palmar-plantar erythrodysesthesia syndrome, plantar erythema, rash, rash erythematous, rash generalized, rash macular, rash maculo-papular, skin atrophy, skin exfoliation, skin fissures, skin lesion, skin ulcer, toxic skin eruption, xeroderma.

## 4. GU Oncology Anticancer Drugs

The following section outlines the various antineoplastic agents currently in use in genitourinary malignancies and outlines the type and frequency of skin eruptions associated with each. Guidelines on dose interruptions and reductions for dermatologic toxicities related to each drug can be found in individual agents’ product monographs. Except for SCARs or other potentially fatal eruptions, a culprit agent can sometimes be continued while supportive and eruption-directed measures are employed (for example, topical corticosteroids) if the severity of the eruption is grade ≤2, though the decision to do so should be made on a case-by-case basis. Patient functional status and preferences, disease tempo and responsiveness to therapy are all factors that should be considered when deciding whether to continue any given systemic therapy while treating dermatologic toxicity. The onset of grade 3 or higher toxicities should prompt discontinuation of any potential culprit agent while the toxicity is addressed.

## 5. Antibody-Drug Conjugates

Antibody-drug conjugates (ADCs) are a rapidly evolving class of therapeutic agents in oncology. Each ADC consists of a monoclonal antibody that binds to a specific molecular target, a cytotoxic payload, and a molecular linker between the two. Upon binding, the compound is internalized by endocytosis and processed by the lysosomal system, which releases the payload in the cellular cytoplasm causing cell death [111].

### Enfortumab Vedotin (EV)

EV is composed of enfortumab, a monoclonal antibody targeting nectin-4, linked to a payload of monomethyl auristatin E (MMAE), a potent anti-microtubule agent. Nectin-4 is an adhesion molecule that is highly expressed in urothelial cancers and human epidermal keratinocytes in the skin [112]. EV is used as a standalone treatment for refractory metastatic urothelial carcinoma (mUC) [113], and is combined with pembrolizumab in the first-line setting [42].

Due to the prevalent expression of Nectin-4 in keratinocytes, skin toxicities associated with EV are common and can be severe. In the EV-301 study, 43.9% of patients treated with EV monotherapy experienced skin reactions of any grade, with 14.5% being grade 3 or higher [113]. While all reactions were termed “rashes”, they were predominantly maculopapular, with about one-fifth further characterized as severe cutaneous adverse reactions, which includes a wide variety of manifestations such as stomatitis, drug eruption, conjunctivitis, dermatitis bullous, skin exfoliation, blister, erythema multiforme, exfoliative rash, fixed eruption, mouth ulceration, pemphigus (see Box 1 and Box 2), flexural exanthema, and toxic skin eruption amongst others [113]. Xerosis and pruritus (see Box 6) are commonly observed with an incidence of 17% and 32%, respectively, in the EV-301 trial [113]. According to the monograph, a pooled analysis of all EV studies shows the median time to onset of severe cutaneous adverse reaction is 0.6 months (ranging from 0.1 to 6.4 months). In patients resuming infusion after drug interruption related to skin toxicity, recurrence was 24% in patients resuming EV at the same dose and 16% in those resuming at a reduced dose [45].

Stevens–Johnson syndrome/toxic epidermal necrolysis (SJS/TEN) (see Box 1) has been reported and associated with fatalities [43]. At least one real-world analysis demonstrates a 15-fold increased risk of SJS/TENS with EV use compared to other chemotherapeutic agents [114]. While the expression of Nectin-4 on epithelial tissue is felt to be the primary mechanism by which EV causes these toxicities, the cytotoxic payload (monomethyl auristatin E) likely plays a role, as other ADCs with MMAE payloads such as brentuximab vedotin can also cause significant skin toxicities. A retrospective analysis found that about one-third of 611 patients with lymphoma treated with brentuximab vedotin experienced dermatologic adverse events with maculopapular rashes being the most common (62%) [115]. Cases of SJS/TENS have also been described in patients receiving brentuximab vedotin [116].

The incidence of dermatologic adverse events associated with EV increases when combined with immune checkpoint inhibitor pembrolizumab (EV-P). In EV-302 trial, the rate of skin toxicity reported with EV-P was as high as 66.8%, with grade ≥ 3 reactions of 15.5% [117]. No deaths related to skin toxicity were reported. Likewise, in Cohort K of the Phase 1/2b EV-103 study, the incidence of skin toxicity of any kind was 67.1% versus 45.2% in the EV-P and EV arms, respectively, with 21.1% of patients in the EV-P arm incurring Grade 3 or worse dermatologic side effects compared to only 8.2% in patients receiving EV monotherapy [45]. Given the relatively high incidence of dermatologic toxicity with EV-P, it is reasonable to assess patients after each EV infusion for the first two cycles of EV-P in order to rapidly identify and manage evolving eruptions.

## 6. Androgen Receptor Pathway Inhibitors (ARPI)

ARPIs are widely used in the treatment of prostate cancer. The four most commonly used agents are the androgen receptor blockers apalutamide, enzalutamide, darolutamide, and the androgen biosynthesis inhibitor abiraterone. While all target the androgen receptor pathway, the incidence and severity of skin toxicity among patients receiving these drugs varies markedly.

### 6.1. Apalutamide

Apalutamide is associated with a high incidence of skin toxicity, ranging from 23.8 to 27.1%, with 5.2 to 6.3% being Grade ≥ 3, as observed in the SPARTAN and TITAN clinical trials [56,57]. Although “rash” in these studies was defined broadly (encompassing maculo-papular rash, urticaria, conjunctivitis, erythema multiforme, skin exfoliation, stomatitis, blister, pemphigoid, and more), case reports and series highlight both common non-life threatening rashes (such as urticaria) [110,118], and potentially fatal SCARs like SJS/TEN and AGEP can occur (see Box 1, Box 2 and Box 3) [119,120]. The time to onset of these rashes tends to be longer than typical drug eruptions, averaging nine weeks according to both Shima et al. and a review from Memorial Sloan Kettering Cancer Center [118,121,122]. In addition to an approach to identifying and treating morbilliform rashes (see Box 1), patient education regarding preventive and supportive measures may be helpful. In a prospective study by Shore et al., patients were given a rash management guide including recommended skin care practices. Rash-related outcomes such as the incidence, severity, and duration of rashes were improved in the study population compared to trial populations in SPARTAN and TITAN [123].

### 6.2. Enzalutamide

Enzalutamide has a relatively lower incidence of skin toxicity, with 0–4% of patients reported in large phase III trials experiencing some form of rash, including maculopapular rash [57,61,62,63]. Nonetheless, case reports of SCARs, such as AGEP [124] and SJS/TEN have been documented [125]. Notably, at least one fatality attributed to enzalutamide-induced TEN has been described [126]. In many cases of SCARs identified in the literature—such as AGEP and SJS/TEN—the onset of symptoms occurred within two weeks of initiating enzalutamide (see Box 1, Box 2 and Box 3).

### 6.3. Darolutamide

Darolutamide is employed either as monotherapy or in combination with docetaxel for prostate cancer treatment [66,127]. Skin toxicity from darolutamide is comparatively rare, with 2.9% of patients in the ARAMIS trial reporting rash of any grade; of these, only once case was classified as Grade 3 [67]. Similarly, the incidence of rash in a trial studying its use in conjunction with docetaxel and androgen deprivation therapy (ADT) was only marginally higher than the comparator arm of docetaxel and ADT (16.6% versus 13.5%, respectively) [127].

### 6.4. Abiraterone Acetate with Prednisone

Abiraterone acetate acts as an irreversible inhibitor of CYP-17, an enzyme involved in the production of dehydroepiandrosterone and androstenedione [71]. It is co-administered with prednisone and can be combined with docetaxel [128]. In contrast to other ARPIs, the rate of skin toxicity is not as high, possibly due to its co-administration with prednisone. No skin reactions were reported in the COU-AA-301 or LATITUDE [70,129], whereas COU-AA-302 reported 8.1% rash and 3.5% other “skin reactions” compared to 3.7% and 0.9% in the placebo arm, respectively [72]. STAMPEDE reported 14% grade 1–2 maculopapular rash, vs. 9% for placebo as well as 25% “other skin disorder” vs. 17% for placebo [72]. One retrospective analysis indicated that rashes could occur with long-term exposure (median of 24 months) [130]. Despite its favorable skin safety profile, cases of SJS/TENS have been reported in FAERS [1].

## 7. Poly ADP Ribose Polymerase (PARP) Inhibitors

PARP inhibitors are oral agents employed in the treatment of prostate cancer, in combination with ADT, with or without ARPI. These inhibitors trap PARP1 and PARP2 with varying degrees of potency, leading to induction of apoptosis in cells with deficiencies in homologous recombination repair pathways. Several PARP inhibitors, including olaparib, rucaparib, niraparib, and talazoparib, have been evaluated as both monotherapy and in combination with ARPIs [76,77,82,84,86]. Olaparib and niraparib have been used in combination with abiraterone, while talazoparib has been used in combination with enzalutamide.

Rucaparib has been linked to higher skin toxicity compared to other PARP inhibitors, specifically photosensitive eruptions. In both TRITON-2 and 3 trials, incidence of photosensitivity was 7–10% and rash occurred in 7–8% of patients, a much higher rate than other PARP inhibitors [80,81]. Patients should be advised to limit exposure to sunlight and use sunscreen or photoprotective clothing. Notably, combination regimens of PARP inhibitors with either abiraterone or enzalutamide do not appear to increase the risk of skin toxicity significantly [76,83,85]. Overall, the incidence of skin toxicity associated with PARP inhibitors is low, photosensitivity seems limited to rucaparib and, accordingly, the number of reports on severe cutaneous reaction very anecdotal. FAERS only contains one case of SJS/TEN with niraparib, and no cases of AGEP or DRESS [1]. Similarly, the FAERS database contains only two cases of olaparib causing pemphigoid skin reactions.

## 8. Immunotherapy

### Immune Checkpoint Inhibitors (ICIs)

ICIs are a class of immunotherapy that prevent immune evasion by malignant tumours, thereby activating cytotoxic immune cells to generate an antitumour response. ICIs commonly used in GU malignancies target programmed cell death protein 1 (PD-1, e.g., pembrolizumab, nivolumab), programmed death-ligand 1 (PD-L1, e.g., durvalumab, avelumab), and cytotoxic T-lymphocyte-associated protein 4 (CTLA-4, e.g., ipilimumab). ICIs are approved for use for RCC and urothelial cancers. They can be used as monotherapy or in combination, either as doublet immunotherapy (e.g., ipilimumab + nivolumab) or with VEGFR-targeting TKIs, ADCs and cytotoxic chemotherapy.

Skin toxicities are common with ICIs, resulting from autoimmune inflammation. Manifestations can include any of the aforementioned morphologies (Box 1, Box 2, Box 3, Box 4, Box 5, Box 6 and Box 7) and more. While these eruptions are usually mild or moderate in severity, they can occasionally progress to life-threatening conditions such as SJS/TEN, AGEP, and DRESS [47]. The rates of any-grade rash in phase III trials of pembrolizumab or nivolumab monotherapy range from 10 to 20%, and pruritus 23%; with grade ≥ 3 rash occurring in less than 1% of cases. In the combination of ipilimumab and nivolumab, any-grade rash was reported at an incidence of 22%, and pruritus 28%; with grade ≥ 3 rash reported at less than 1%. Symptoms can arise within weeks of initiating therapy and up to several months after discontinuation [131].

The incidence of dermatologic side effects appears to be independent of the treatment line [101,106]. Comprehensive guidelines such as those published by ESMO and Cancer Care Ontario (CCO) provide valuable frameworks for the diagnosis and management of checkpoint-inhibitor related skin toxicity [132,133].

## 9. Tyrosine Kinase Inhibitors (TKIs)

TKIs are oral small molecules targeting multiple kinases, including VEGFR, MET, and FGFR. TKIs that predominantly inhibit VEGFR are used in the treatment of metastatic or unresectable RCC. Notable examples of these inhibitors in GU oncology include sunitinib, cabozantinib, pazopanib, lenvatinib, axitinib, and tivozanib [134]. Many TKIs are used in combination with immune checkpoint inhibitors or are being actively investigated in such contexts. Additionally, erdafitinib, an FGFR1-4 inhibitor, has demonstrated clinical efficacy in patients with FGFR 2/3-altered locally advanced or metastatic urothelial carcinoma [100,135].

VEGFR-targeting TKIs are associated with a characteristic set of skin toxicities, including maculopapular eruptions and PPE [136]. PPE is particularly notable as it is a hallmark adverse effect of this drug class. In the frontline treatment of RCC, the prevalence of PPE ranges from approximately 30–50%, regardless of the TKI used, or whether it is used in monotherapy or in combination with checkpoint inhibitor immunotherapy [90,91,92,93]. In contrast, the incidence of PPE with erdafitinib in patients with metastatic or unresectable urothelial cancer is comparatively lower, at 22 to 23% [100,135]. Given that PPE has been a potential side effect of systemic treatment for decades, the detailed discussion of its diagnosis and management is beyond the scope of this article; however several comprehensive reviews are available on this topic and the treatment outline is presented in Box 8 [137,138]. It is worth noting that reports of DRESS and SJS/TEN have been reported with most of these TKI in the FAERS dashboard (see Table 1) [1].

## 10. Hypoxia-Induced Factor (HIF)-2α Inhibitor

### Belzutifan

Belzutifan, a hypoxia-induced factor (HIF)-2α inhibitor indicated for the treatment of non-metastatic von Hippel Lindau-associated RCC and demonstrated efficacy in refractory metastatic RCC [104]. It is also under investigation in combination with immune checkpoint inhibitors, and TKIs. In the phase III LITESPARK-005 trial, skin toxicity with belzutifan monotherapy was infrequent, with rashes occurring in 4.6% of patients [104]. In the phase II LITESPARK-003 trial, which assessed belzutifan in combination with cabozantinib, rashes were reported in 15.3% of patients (8 out of 52), with five cases being Grade 1. There were no cases of Grade 3 or higher skin toxicity [139].

## 11. Mechanistic Target of Rapamycin (mTOR) Inhibitors

### Everolimus

Everolimus inhibits the mTOR serine/threonine kinase, a critical regulator of cellular growth, proliferation, and survival. In the phase III RECORD-1 trial, which evaluated everolimus in patients with refractory metastatic RCC, rash was reported in 29% of patients, though less than 1% experienced grade 3 or higher severity [140]. More recently, in the phase 3 CLEAR trial, the combination of everolimus and lenvatinib was associated with skin toxicity, with 24.8% of patients developing rashes (mostly grade 1–2). Additionally, PPE was reported in 22.8% of patients, (2.8% ≥ grade 3), in line with lenvatinib’s known toxicity profile. SJS/TEN, DRESS, and AGEP have been reported on the FAERS dashboard [1].

## 12. Discussion

Our work provides clinicians with a practical framework for identifying, categorizing, and managing skin toxicities associated with newer therapies in GU oncology, aiming to improve patient care in this rapidly evolving field.

It is critical for oncologists to appreciate the fact that the occurrence of dermatologic toxicity does not preclude re-challenge with a given agent. If patients’ initial eruptions are managed with dose interruption, supportive care, or directed treatment (e.g., topical or systemic corticosteroids), re-challenge can be attempted in most cases at either the same or a reduced dose. The major exceptions are severe or life-threatening reactions such as SJS/TENS, DRESS, and AGEP, where re-challenge is not recommended. Dermatology consultations can be helpful in identifying eruption type and etiology, assisting with symptom management and optimizing systemic therapy rechallenge.

The above synthesis outlined in Table 1, and our review of the literature, highlight a major limitation of this field and the urgent need for better reporting of dermatologic toxicities. The most commonly reported dermatologic side effect in Table 1 is “rash”. Unfortunately, we are of the opinion that this term does not provide enough information to be useful for most practicing clinicians. Furthermore, many of the changes noted that are not classified as “rash” still do not offer an accurate diagnosis (e.g., drug eruption, papule, macule, etc.). This limitation in the literature has been compounded by many retrospective studies examining skin toxicities with cancer treatments, where rash or descriptive terms are used instead of specific diagnoses [141,142,143,144]. Indeed, these authors may be being cautious, trying to avoid anchoring certain diagnoses to a specific drug too early. “Psoriasis-like rash”, as described in L’Orphelin’s retrospective series, is still more helpful than other less specific terms. The existing literature includes publications attempting to report different specific reactions, however, oftentimes this is limited to case series without a wider context to provide a risk profile for patients receiving the drug [20,145,146]. Although time course is likely most helpful, without this wider context it is difficult for the practicing clinician to discern if the skin changes they are attempting to treat are idiopathic or iatrogenic.

## 13. Conclusions

The evolving treatment landscape in genitourinary oncology has introduced an array of novel systemic therapies, each accompanied by unique skin toxicities. These skin adverse events, although usually manageable, can significantly impact treatment continuity and quality of life if not properly addressed. Our review highlights the spectrum of cutaneous drug reactions associated with the various drug used in GU oncology. The complexity of managing these reactions is heightened by the use of combination therapies, which can present overlapping or more severe side effects.

By improving the specificity and accuracy of dermatologic side effect reporting in clinical trials, and fostering closer collaboration between oncologists and dermatologists, we can enhance patient outcomes. Continued research is essential to develop standardized guidelines for managing these skin toxicities, ensuring that patients can tolerate and benefit from these treatments without unnecessary interruptions.

Future research into dermatologic toxicity from cancer treatment should aim to provide more specific diagnoses. These data would help medical oncologists provide more targeted toxicity management as well as make more informed decisions about patients’ systemic therapy.

## Supplementary Materials

The following supporting information can be downloaded at: https://www.mdpi.com/article/10.3390/cancers17020251/s1. FDA adverse event reporting system query for SJS/TEN, AGEP, DRESS and pemphigoid crossmatched with all GU oncology drugs.

## Appendix A. CTCAE Grading of Common and Significant Dermatologic Toxicities [111]

**Dermatologic Toxicity**	**Grade 1**	**Grade 2**	**Grade 3**	**Grade 4**	**Grade 5**
**Acneiform Rash**	Papules and/or pustules covering <10% BSA, which may or may not be associated with symptoms of pruritus or tenderness	Papules and/or pustules covering 10–30% BSA, which may or may not be associated with symptoms of pruritus or tenderness; associated with psychosocial impact; limiting instrumental ADL; papules and/or pustules covering >30% BSA with or without mild symptoms	Papules and/or pustules covering >30% BSA with moderate or severe symptoms; limiting self-care ADL; associated with local superinfection with oral antibiotics indicated	Life-threatening consequences; papules and/or pustules covering any % BSA, which may or may not be associated with symptoms of pruritus or tenderness and are associated with extensive superinfection with IV antibiotics indicated	Death
**Bullous Dermatitis**	Asymptomatic; blisters covering	Blisters covering 10–30% BSA; painful blisters; limiting instrumental ADL	Blisters covering >30% BSA; limiting self care ADL	Blisters covering >30% BSA; associated with fluid or electrolyte abnormalities; ICU care or burn unit indicated	Death
**Dry Skin**	Covering <10% BSA and no associated erythema or pruritus	Covering 10–30% BSA and associated with erythema or pruritus; limiting instrumental ADL	Covering >30% BSA and associated with pruritus; limiting self care ADL	Not Applicable	Not Applicable
**Eczema**	Asymptomatic or mild symptoms; additional medical intervention over baseline not indicated	Moderate; topical or oral intervention indicated; additional medical intervention over baseline indicated	Severe or medically significant but not immediately lifethreatening; IV intervention indicated	Not Applicable	Not Applicable
**Erythema Multiforme**	Target lesions covering <10% BSA and not associated with skin tenderness	Target lesions covering 10–30% BSA and associated with skin tenderness	Target lesions covering >30% BSA and associated with oral or genital erosions	Target lesions covering >30% BSA; associated with fluid or electrolyte abnormalities; ICU care or burn unit indicated	Death
**Maculopapular Rash**	Macules/papules covering <10% BSA with or without symptoms (e.g., pruritus, burning, tightness)	Macules/papules covering 10–30% BSA with or without symptoms (e.g., pruritus, burning, tightness); limiting instrumental ADL; rash covering >30% BSA with or without mild symptoms	Macules/papules covering >30% BSA with moderate or severe symptoms; limiting self care ADL	Not Applicable	Not Applicable
**Pruritus**	Mild or localized; topical intervention indicated	Widespread and intermittent; skin changes from scratching (e.g., edema, papulation, excoriations, lichenification, oozing/crusts); oral intervention indicated; limiting instrumental ADL	Widespread and constant; limiting self care ADL or sleep; systemic corticosteroid or immunosuppressive therapy indicated	Not Applicable	Not Applicable
**Stevens** **–Johnson Syndrome**			Skin sloughing covering <10% BSA with associated signs (e.g., erythema, purpura, epidermal detachment, and mucous membrane detachment)	Skin sloughing covering 10–30% BSA with associated signs (e.g., erythema, purpura, epidermal detachment, and mucous membrane detachment)	Death
**Toxic Epidermal Necrolysis**				Skin sloughing covering >=30% BSA with associated symptoms (e.g., erythema, purpura, or epidermal detachment)	Death
